# Delayed breast cancer presentation: hospital data should inform proactive primary care

**DOI:** 10.4102/phcfm.v5i1.503

**Published:** 2013-08-15

**Authors:** Tessa S. Marcus, Samy Lunda, Leticia Fernandez

**Affiliations:** 1 Department of Family Medicine, University of Pretoria, South Africa; 2 Family Physician, Sebokeng Hospital, South Africa

## Abstract

**Background:**

Breast (and cervical) cancer affects a growing proportion of women in South Africa. Although treatable, where health literacy is low, women typically seek medical attention only when their condition is at an advanced stage and difficult to contain.

**Objectives:**

To understand the sociodemographic characteristics of women who present with advanced breast cancer in order to intervene proactively in primary care.

**Method:**

A retrospective analysis of women with advanced breast cancer (Stage IIb and higher) at a Level 2 regional hospital in South Africa (2007–2010).

**Results:**

The average age amongst the 103 women enrolled in this study was 59. One-third of the women had secondary education, 35% were unemployed and two-thirds were not married. Nearly 11% (*n* = 11) of the women had previously had cancer. Lumps (*n* = 87) were the most common reason for seeking healthcare and were, together with axillary lymph node abnormalities (84.5% and 19.4% respectively), the most common clinical symptoms. Symptoms were noticed by 52% (*n* = 54) of the women more than six months prior to their first consultation. A personal history of cancer increased *threefold* the odds of presenting within three months. Middle-aged women were twice as likely as those < 45 and > 65 to report within three to six months. Secondary education increased the odds of presenting within three to six months by 56%. Employment and marital status were not significant.

**Conclusion:**

The women most at risk for delayed detection and treatment were those without a history of breast cancer, aged < 45 and > 65, with low education. They can best be reached through low-cost community-orientated primary care that proactively provides health education and promotes self- and clinical examination at the individual, family, clinic and general practitioner level.

## Abstrait

**Retard de détection du cancer du sein: les données des hôpitaux devraient informer une prise en charge primaire proactive**

**Contexte:**

Le cancer du sein (et du col de l'utérus) affecte une proportion croissante de femmes en Afrique du Sud. Bien que soignable, lorsque les informations sanitaires sont insuffisantes, les femmes ne consultent généralement que lorque leur état est à un stade avancé et difficile à contenir.

**Objectifs:**

Comprendre les caractéristiques sociodémographiques des femmes qui présentent un cancer du sein avancé afin d'intervenir de manière proactive dans la prise en charge primaire.

**Méthodes:**

Analyse rétrospective des femmes souffrant d'un cancer du sein avancé (Stade IIb et plus) dans un hôpital régional de niveau 2 en Afrique du Sud (2007–2010).

**Résultats:**

L’âge moyen des 103 femmes inscrites à cette étude était de 59 ans. Un tiers des femmes avaient suivi l'enseignement secondaire, 35% étaient sans emploi et les deux tiers n’étaient pas mariées. Près de 11% (*n* = 11) des femmes avaient déjà eu un cancer. Des grosseurs (*n* = 87) constituaient la raison les plus courante de recherche de soins de santé et étaient, avec les anomalies de ganglions lymphatiques axillaires (84,5% et 19,4% respectivement), les symptômes cliniques les plus courants. Les symptômes ont été remarqués par 52% (*n* = 54) des femmes, plus de six mois avant leur première consultation. Des antécédents personnels de cancer mutlipliaient *par trois* les chances de se présenter dans les trois mois. Les femmes d’âge moyen avaient deux fois plus de chances que celles de < 45 ans et de > 65 ans de se présenter dans les trois à six mois. L’éducation secondaire augmentait de 56% les chances de se présenter dans les trois à six mois. Les situations professionnelle et conjugale n’étaient pas significatives.

**Conclusion:**

Les femmes courant le plus de risque d'une détection et d'un traitement retardés étaient celles sans antécédents de cancer du sein, dont l’âge était < 45 ans et > 65 ans, et ayant peu d’éducation. Elles peuvent être atteintes par le biais d'une prise en charge primaire à faible coût et orientée sur la communauté, qui fournit de manière proactive une éducation en santé et encourage l'auto-examen et l'examen clinique au niveau individuel, familial, clinique et du généraliste.

## Introduction

With over 1.3 million women worldwide diagnosed with breast cancer every year,^1^ it can be inferred that there is no part of the world where this disease is rare and no female population where the risk of breast cancer is truly low.^2^ The incidence of breast cancer is presently fourfold higher in the high-income countries of North America and Europe as compared with the middle- and low-income countries of Asia, Africa and Latin America. This picture is expected to change within the next decade or two, by which time it is expected that 70% of cancers detected will be in low- and middle-income countries.^3^

Clinical advances in the treatment of breast cancer during the past half century have led to major improvementments in health outcomes including longer disease-free survival, less surgical mutilation and increasing individualisation of treatment.^4^ In addition to improved localised cancer control, successful treatment of regional disease with nodal involvement has also made a small dent on late stage mortality, at least in the United States (US). Over the 30 years spanning 1978 to 2008, mortality from breast cancer amongst women over 40 years of age decreased by 28% in the US.^5^ More generally, five-year survival rates are consistently over 70% in upper- and middle-income countries.^6^

Concomitant with advancements in treatment, there has been a concerted effort to raise public understanding with regard to the importance of early detection in and primary care for women. Together, these have led to the steady down-staging of breast cancer in more developed countries. Notable recent research in the US suggests that the availability of screening mammography has played a lesser role in this outcome, given that the most significant decrease in breast cancer death rates (42%) was amongst women below the age of 40 who were not routinely exposed to the technology.^6^

In sharp contrast, female mortality rates from breast cancer are greatest amongst the poor and in low- and middle-income countries. There, breast cancer accounts for 55% of cancer-related deaths annually^6^ and they are expected to increase by more than 50% again over the next decade. Five-year survival rates in low- and lower-middle income countries range widely from as low as 12% in the Gambia to around 47% in Egypt.^3^ These outcomes reflect inter- and intra-country social inequalities as well as healthcare system inequities. In contexts such as South Africa, where the disease burden is high, breast- and other cancers compete for attention with infectious and non-communicable chronic diseases such as HIV, tuberculosis, hypertension, cardiovascular disease and diabetes. These limitations are compounded by healthcare systems that are hospital-centric and disjointed. They often fail to meet the basic diagnostic and care requirements of the people entering them, thereby compounding delays in detection and treatment whilst adding to out-of-pocket patient costs. They also fail to mobilise the necessary personnel and financial resources required to engage promotion and prevention proactively with people in their homes and workplaces. In South Africa, concern about the structure and functioning of the healthcare system itself has led to a national effort to re-engineer primary care.^7^

Additionally, high rates of breast cancer mortality are the product of low levels of health literacy, gender inequalities and delayed individual health-seeking behaviour. Women invariably seek medical help when tumours are large or their cancers are incurable. For example, in South Africa, over 60% of women present with tumours larger than 5 cm.^8^ Also, women with breast cancer present with advanced localised or metastasised cancer (Stages III and IV) in Malaysia (30% – 40%) and Egypt (70%) respectively.^3^ In general, patients with *total* delays of three to six months are known to have significantly worse survival than those with delays of less than three months.^9^

The reasons women with possible breast cancer delay in seeking healthcare are also influenced by demographic, socioeconomic, clinical and psychological factors.^2^ According to a systematic review of available world literature on patient-mediated time and stage delay in common cancers,^10^ delay is associated with patient age and levels of education and symptom type in breast cancer. Older age and lower levels of education were found to increase the risk for delayed presentation, as were atypical symptoms that did not include a lump. Marital status was found to be unrelated to patient presentation patterns. Also, intensive instruction in breast self-examination through teaching examination skills and providing public health information was not found to impact positively on breast cancer mortality, in and of itself.^11^

This paper presents the findings of a study of the socio-demographic profile of women with advanced breast cancer who delayed presentation at a regional hospital in South Africa. The findings are interpreted against the backdrop of re-engineered primary care,^7, 12^ modelled on Community-Oriented Primary Care (COPC).^13^

## Significance of work

Breast (and cervical) cancer is nested in the quadruple burden of disease as a key condition that affects a growing proportion of women in South Africa. Although treatable, with good clinical prognosis if detected early, women in low- and middle-income countries typically seek medical attention when their condition is at an advanced stage and difficult to contain. This also holds true for South Africa. Through a retrospective study of patient records at a Level 2 Regional hospital in Gauteng, South Africa, the documented socioeconomic and demographic characteristics of women who delay in presenting with advanced breast cancer at the facility provide us with a more refined profile of women at risk of detection and treatment delay. These findings, in turn, can assist in the direction of primary care interventions around breast cancer awareness, self-examination and low cost, proactive clinical examination at a general practitioner, clinic, community and family level.

## Ethical considerations

The study (protocol 87/2011) was approved by the University of Pretoria, Main Research Ethics Committee. It was also approved by the Chief Executive Officer of Sebokeng Hospital and the Ethics Committee of Gauteng Province. Patients were not directly involved in the research and therefore did directly benefit nor were they put at any risk by the study. Patient records were blinded before being accessed in order to preserve patient anonymity.

## Research method and design

This retrospective descriptive study was based on a file review of patients who presented with advanced breast cancer (Stage IIb or higher) at the Sebokeng Hospital Breast Clinic between January 2007 and December 2010.

Sebokeng Hospital is a regional facility located in Sedibeng Municipal District, at the heart of the densely-populated, mining industrial complex of south Gauteng. It is a level 2 public hospital that serves some 800 000, mostly African, people. Many (35%) are unemployed. Of those who work, most are self-employed or employed in manufacturing, trade and transport and community service.^14^

Sebokeng Hospital is a multi-disciplinary facility combining general specialist disciplines such as Internal Medicine, Surgery, Paediatrics, Gynaecology and Emergency Care. It serves as a referral facility for 10 primary health clinics and community health centres. Sebokeng Hospital also runs its own breast clinic that can be accessed without referral. At the time of the study, Sebokeng Hospital did not have a mammography machine. As a consequence, patients in need of a screening mammogram had to be transported 50 km to Chris Hani Baragwanath Hospital for screening. Screening results were sent back to Sebokeng Hospital Breast Clinic and patients with breast cancer were then referred for treatment and follow up to Charlotte Maxeke Johannesburg General Hospital, over 60 km away.

### Patients

The records of all patients presenting with advanced breast cancer at Sebokeng Hospital from January 2007 until December 2010 were reviewed and then analysed in terms of sociodemographic data and clinical information. Patients included in the study had Stage IIb or higher advanced breast cancer at the time of first presentation at Sebokeng Hospital Breast Clinic. Patients with early-stage breast cancer were excluded. Stage IIb refers to invasive breast cancer where the tumour is 2 cm – 5 cm and has spread to the axillary lymph nodes or the tumour is larger but has not yet spread to the axillary lymph nodes. Stage III involves invasive breast cancer tumours of any size that have spread to the axillary lymph nodes, the chest wall and/or the skin of the breast. Stage IV describes cancer that has spread (metastasised) to other organs (lungs, liver, brain and skin) and/or to more distant lymph nodes.^8^

The time between patient-reported onset of symptoms and initial presentation at the Sebokeng Hospital Breast Clinic was collected in order to determine the length of patient delay. Although we are cognisant of possible recall issues regarding reported onset of symptoms, we have no way of assessing the accuracy of this information, which is routinely collected during history taking. Stage of cancer was based on clinical information as well as pathology results. Self-reported demographics (age and marital status), socioeconomic (education and employment status) and clinical (history of cancer) data were extracted from the patient records.

### Data analysis

Delay in presentation by patients was defined as waiting for three or more months from patient-reported onset of initial symptoms to first medical consultation at the facility.^9^ Descriptive statistics, measures of association between patient characteristics and delays to presentation, as well as multivariate analysis, were carried out using Stata data analysis and statistical software (v.9).

## Results

The findings in this study are based on the patient records of 103 women presenting with advanced breast cancer at the breast clinic. Table 1 shows the sociodemographic characteristics of these women, including their ages. The mean age of the women in the sample was 59 (range 34–83), with two thirds aged 55 or older.

**TABLE 1:** Sample characteristics: Patients presenting with breast cancer.

Variables	Patients (*n* = 103)	%
**Age†**		
34–44	10	9.7
45–54	25	24.3
55–64	36	35.0
65–83	32	31.1
**Education**		
No school	30	29.1
Primary school	37	35.9
Secondary school or higher	36	35.0
**Employment status**		
Not employed	36	35.0
Employed	67	65.0
**Marital status**		
Single/Divorced/Widowed	69	67.0
Married	34	33.0
**Patient's history of cancer**		
Yes	11	10.7
No	92	89.3
**Patient's delay in presenting to clinic (from self-reported date of first symptoms)**		
< 3 months	18	17.5
3 to 6 months	31	30.1
> 6 months	54	52.4
**Stage of cancer at presentation to clinic**		
IIB	5	4.9
IIIA to IV	98	95.1

† Average age = 59.1.

In terms of education, 29% of the women (*n* = 30) had never attended school. The remainder were almost evenly split between those with primary (*n* = 37) and those with secondary (*n* = 36) education. 35% (*n* = 36) were unemployed and over two-thirds (*n* = 69) described themselves as not being married at the time they first presented at the Sebokeng Hospital Breast Clinic.

Nearly 11% (*n* = 11) had previously had some form of cancer. More than half (52%, *n* = 54) reported that they had first noticed symptoms six or more months prior to the initial consultation. Lumps (84.5%) were the most common self-identified symptoms. Eighty-seven women in the sample reported it either as their only symptom or as being in combination with another symptom. Most of the cancers (95%, *n* = 98) were deemed clinically to be Stage III or higher at presentation.

Table 2a and b show that for the majority of women (71%, *n* = 73), only one symptom was recorded at the time of first presenting with breast cancer, 28% (*n* = 29) had two symptoms and only one woman reported three symptoms (breast lump, skin change and axillary lymph node abnormalities). Breast lumps (84.5%) (*n* = 87) and axillary lymph node abnormalities (19.4%) (*n* = 20) were the most common clinical symptoms.

**TABLE 2a:** Breast cancer symptoms at first presentation to clinic.

Symptoms†	Number of women	%
Breast lumps	87	84.5
Axillary node abnormal	20	19.4
Abscess/ulcers	8	7.8
Nipple discharge	7	6.8
Skin changes	7	6.8
Pain	5	4.9

† Recorded in patients’ file alone or in combination with other symptoms. Adds to more than the number of patients in the study because patients may have reported more than one symptom.

**TABLE 2b:** Breast cancer symptoms at first presentation to clinic

Number of symptoms at first presentation to Clinic†	Number of women	%
One symptom alone	73	70.9
Two symptoms	29	28.1
Three symptoms	1	1.0

† From medical files.

Table 3 shows the bivariate association between delay in presentation and various sociodemographic and clinical variables in the analysis. These associations are further examined later on using multivariate analysis. Through bivariate analysis, the data show that delays in presentation varied with age. Waiting six or more months before seeking healthcare was more likely amongst women in the youngest (ages 34–44) and oldest (ages 65–83) groups than amongst those in the middle-aged group (ages 45–64). The reasons for this pattern would need to be investigated further.

**TABLE 3:** Bivariate associations with delays in presenting to Clinic for breast cancer symptoms.

Variables	% of women in each row category who presented to clinic to report symptoms for the first time at:								Pearson's Chi^2^(df)
		
	< 3 months		3–6 months		> 6 months		All		
					
	*n*	%	*n*	%	*n*	%	*n*	%	
**Age**									
34–44	3	30.0	1	10.0	6	60.0	10	100	Chi^2^(6) = 11.5*
45–54	4	16.0	11	44.0	10	40.0	25	100	
55–64	7	19.4	14	38.9	15	41.7	36	100	
65–83	4	12.5	5	15.6	23	71.9	32	100	
*Average age (years)*	*18*	*55.7*	*31*	*56.6*	*54*	*61.7*	103	*-*	Chi^2^(2) = 9.5**
**Education**									
No school	3	10.0	5	17.7	22	73.3	30	100	Chi^2^(4) = 10.6**
Primary school	6	16.2	11	29.7	20	54.1	37	100	
Secondary school or higher	9	25.0	15	41.7	12	33.3	36	100	
**Employment status**									
Not employed	4	11.1	6	16.7	26	72.2	*36*	100	Chi^2^(2) = 8.7***
Employed	14	20.9	25	37.3	28	41.8	*67*	100	
**Marital status**									
Single/Divorced/Widowed	11	15.9	16	23.2	42	60.9	69	100	Chi^2^(2) = 6.4**
Married	7	20.6	15	44.1	12	35.3	34	100	
**History of cancer**									
No	9	9.8	29	31.5	54	58.7	92	100	Chi^2^(2) = 36.2***
Yes	9	81.8	2	18.2	0	0.0	11	100	

* Denotes differences between groups that are statistically significant at *p* < = 0.10;

** *p* < = 0.05;

*** *p* < = 0.01.

In terms of education, employment and marital status, women in the most vulnerable circumstances were most likely to delay in seeking healthcare. Amongst women with no schooling, 73% (*n* = 22) presented after six months of noticing symptoms, compared with 54% (*n* = 20) of the women with primary education and 33% (*n* = 12) of those with secondary school education. Of the women who reported being unemployed, 72% (*n* = 26) had delayed coming to the breast clinic for six or more months after initial symptoms, whereas some 42% (*n* = 28) of the employed women had delayed for the same length of time. Of the 69 women who said they were single, separated or divorced, 61% (*n* = 42) delayed presentation for six or more months, compared with 35% (*n* = 12) of the 34 women who reported being married.

Table 3 also shows that patients who had previously had cancer were much more likely to present early for medical care compared with women with no history of cancer. These differences are noteworthy: nine of the 11 women (82%) with a history of cancer presented for medical consultation within three months of first noticing symptoms, with the balance (18%, *n* = 2) reporting within six months. In contrast, only nine of the 92 women (10%) with no history of cancer presented in the first three months. 32% (*n* = 29) did so within three to six months and most (59%, *n* = 54) waited more than six months after first noticing their symptoms.

Table 4 shows the results of multinomial logistics regression models. These examine the influence of each demographic and socioeconomic variable whilst simultaneously taking into account the other variables. We divided the analysis into two parts. Firstly, we examined the characteristics associated with presenting for medical consultation within less than three months from first noticing symptoms versus waiting three to six months. Secondly, we examined factors associated with presenting within three to six months from first noticing symptoms versus waiting more than six months. Our rationale for this approach is that the variables that influence delays in presentation may differ between delay periods and dichotomising the data into less than six months and more than six months may mask these important relationships.

**TABLE 4:** Odds ratios of delays in presenting to Clinic for breast cancer symptoms.

Variables	Odds of patients presenting to clinic to report symptoms:	
	
	Less than 3 months vs.3–6 months delay	3–6 months vs. more than 6 months delay
**Patient's age**		
34–45	-	-
45–54	-1.27	2.05*
55–64	-1.20	2.55**
65–83	0.50	2.28
**Education**		
No schooling	-	-
Primary school	0.23	0.27
Secondary school or higher	1.76	1.56*
**Employment**		
Not employed	-	-
Employed	-0.58	0.63
**Marital status**		
Single/Divorced/Widowed	-	-
Married	-0.48	0.84
**Patient's cancer history**		
No	-	-
Yes	3.57***	22.13***
Constant	-0.89	-4.12

**Pseudo R2**	**0.2578**	
**Number of observations**	***n*** **= 103**	

* Coefficients statistically significant at *p* < = 0.10;

** *p* < = 0.05;

*** *p* < = 0.01.

-, Patient characteristics the reference category.

The second column in Table 4 shows the relative risk ratios of presenting within three months versus waiting three to six months after first symptoms are noticed. We find that having a history of cancer increases *threefold* the odds of presenting to the hospital within three months. No other factor influences timing of presentation between less than three months and three to six months. In contrast, the last column in Table 4 shows that age and education, in addition to a history of cancer, are associated with reporting within three to six months rather than waiting for six months or more. In particular, middle-aged women (45–64) are twice as likely to report within three to six months than younger (under 45) and older (over 65) women. Also, the odds of presenting within three to six months are 56% higher for women with at least some secondary education compared with women with only primary or less schooling. Having had cancer in the past multiplies substantially the odds of presenting within three to six months. It is noteworthy that once age, education and history of cancer are taken into account, employment and marital status are no longer significant predictors of delays in presentation.

## Trustworthiness

This study was conducted using hospital retrospective records which were created by examining clinicians, and whose diagnosis of cancer stage at patient presentation as well as the patients’ sociodemographic data were confirmed several times in the course of subsequent consultations and treatment. The data presented and analysed by the authors are thus credible and dependable and can be verified.

In preliminary analysis the authors obtained similar results to variations in the statistical models, thus the findings reported are deemed to be robust to model specification, reliable and replicable.

## Discussion

In general, our findings show that women with no history of cancer, with low levels of schooling and who are under 45 or over 65 years of age are most at risk of delaying consultation after they first notice symptoms. The risk for patient-delayed presentation with breast cancer is lower for women who have had cancer in the past, have some secondary schooling and/or are aged between 45 and 64 years. Most importantly, all the women with a history of cancer in this sample presented less than six months from first symptoms. Whilst the women in this study were socially- and economically disadvantaged, age and education rather than employment status seemed to determine delayed presentation. Our data suggest that women with low education may both delay presentation and be unemployed****.

Two critical issues stand out from these findings. The first is that direct exposure to cancer makes women more proactive about seeking medical attention in the current healthcare system. Since such exposure is relatively uncommon, it is unlikely to be sufficient to make unexposed women more aware of their risk for breast cancer without specific promotion and prevention efforts. In South Africa and other middle- and low-income countries, women who have had previous exposure to breast cancer are an often underutilised resource in the efforts to raise breast cancer awareness in their communities.

Failure to recognise the potential of cancer survivors to impact on health literacy and health practice in their communities links into the second issue, namely, the limitations of a passive, hospital-centred approach to breast cancer in a context of low health literacy. By its very structure, this approach is unlikely to reach the younger women who may believe that youth is protective of breast cancer, in and of itself. Nor is it likely to reach older women who, in addition to low levels of health literacy, are likely to be constrained by multiple morbidities, additional social burdens, affordability and low health and wellbeing expectations.

The low self-detection of lumps at an early stage, when better prognosis is likely, confirms that breast self-examination is uncommon in the general population. The discovery of lumps (at a late stage) is consistent with but qualifies the international literature that suggests common symptoms, especially lumps, trigger health-seeking behaviour. Clinical breast examination (CBE), as an effective standardised screening examination of the breast, chest wall and axillae^1^ by healthcare practitioners, is also known to *not* be part of routine clinical practice in primary care in South Africa and many other parts of the world.^14, 15^

Without early detection, advances in treatment are likely to have little impact on morbidity and mortality. Proactive self-examination and CBE are low-cost and effective ways of supporting early detection, treatment and follow up. Underlying such practices, however, are assumptions about agency as well as point-of-care responsiveness. They are not simply resolved through self-examination skills training and messaging by or making CBE available in a facility-centred system.^16^ Findings such as these are generated at a regional hospital, a level of healthcare that is poorly situated to reach women when their cancer is at an early stage. Their application runs the risk of being condemned to impotent declaration since these issues have to be addressed elsewhere in the health system by other practitioners amongst sections of the population who are beyond their reach. Clearly, the place to provide breast cancer education and practice, including self- and clinical examination, is at a community level through primary care.

In South Africa, the national drive to re-engineer primary care provides clinicians and other healthcare providers with an opportunity to transform the sociodemographic issues surrounding delayed presentation of breast cancer from sterile, background information into proactive practice. In particular, the adoption and development of COPC as a service-delivery paradigm makes it possible to produce better responses to health outcomes for ordinary women, even where there is multiple morbidity, overstretched systems and underserved populations.^17^ This is especially the case when COPC is supported by a technology-enabled information and communication (ICT) system. Through it, it is possible to use individual health status-assessment data to determine the size of the population of women at *most* risk for delayed breast cancer presentation (< 45 or > 65, with primary or less schooling) in geographically-defined communities (HF Kinkel, personal communication).

In general primary care conditions, where relations are facility centred and systematic ways of ensuring contact are absent, it is difficult to translate generalised information into targeted intervention. With ICT-enabled COPC, it is possible to overcome this difficulty as individual community health workers are linked to and develop relationships with individuals and families in specific, geographically-defined places.^7^ As such, they are able to go to the women identified as being at greatest risk of breast cancer treatment delay, raise their awareness of the risks of breast cancer, educate and support the practice of breast self- and clinical examination and, more generally, develop cancer and health literacy. Where cancer is detected and treated, COPC also enables follow up and treatment support, as it is organised on the principles of both continuity of care and comprehensive care. Once those in most need of early intervention are supported, capacity and priorities permitting, it is also possible to make such an intervention standard primary care practice across entire communities.

## Limitations of the study

This study has several limitations. Methodologically, this is a retrospective study of patient records at a single facility. Data in routine settings is often of moderate quality. Moreover, patient records do not enable in-depth exploration of sociodemographic influences on patient health-seeking behaviour. In particular, they do not allow for the exploration of spouse or household income sources and levels and the general level of education of patients’ families. Study findings are also limited by sampling. In excluding women with early-stage breast cancer at first presentation from the study sample, we are unable to compare the characteristics of women with < Stage IIb with those who first present with more advanced cancer. A third limitation of this study is the absence of information about important individual and community variables, especially study patients’ general health-seeking behaviour prior to their first visit to Sebokeng Hospital Breast Clinic, the distance between patients’ homes and the study hospital as well as the associated transportation costs and availability implications that may have an impact on patient delays in seeking healthcare.

## Conclusion

Patients with advanced breast cancer who delay seeking diagnosis at a regional hospital tend to be either younger or older women (aged < 45 and > 65) without a history of breast cancer and with little education. Early detection and treatment and, with it, the potential to reduce breast cancer mortality in South Africa, is beyond the remit of regional hospital practice. Rather, to achieve these goals it is necessary to reach the women at greatest risk for delayed presentation with breast cancer in their homes and communities. The re-engineering of primary care, operationalised through COPC, provides us with the opportunity to study how proactive support for breast cancer prevention and early detection through community health team supported self- and clinical breast cancer examination can impact on early presentation.
